# Honokiol Suppresses Stemness and Sensitizes Triple-Negative Breast Cancer to Chemotherapy via YAP/TAZ-TEAD Inhibition

**DOI:** 10.32604/or.2026.084576

**Published:** 2026-09-14

**Authors:** Jiang-Nan Xia, Shan-Dong Zhu, Wei-Ling Qu, Yi-Lin Hu, Wen-Yi Ma, Wenyan Wang, Qian-Lan Huang, Bing-Yuan Lin, Jia-En Guo, Ying-Wei Li

**Affiliations:** Research Center of Integrative Medicine, School of Basic Medical Sciences, Guangzhou University of Chinese Medicine, Guangzhou, China

**Keywords:** Honokiol, triple-negative breast cancer, YAP/TAZ–TEAD complex, chemoresistance, cancer stem cells

## Abstract

**Objectives:** As an aggressive subtype of breast cancer, triple-negative breast cancer (TNBC) is constrained by the limited availability of effective treatments and the absence of well-validated therapeutic targets. This study aimed to explore whether honokiol, a potent YAP/TAZ inhibitor, suppresses stem cell–like properties and enhances chemotherapeutic efficacy in TNBC by blocking YAP/TAZ–TEAD transcriptional complex. **Methods:** Through both *in vitro* and *in vivo* models of TNBC, the current study examined how honokiol influences cell proliferation, cancer stem cell (CSC) traits, and paclitaxel sensitivity. To uncover the molecular mechanisms, we analyzed the transcript levels and protein abundance of core YAP/TAZ–TEAD pathway members, including YAP, TAZ, TEADs, ANKRD1, and CYR61. Furthermore, we conducted immunofluorescence staining to examine the nuclear localization of YAP/TAZ. Potential direct interactions were identified using molecular docking, which also predicted the binding affinity between honokiol and the TAZ–TEAD complex. **Results:** Honokiol markedly suppressed the viability and stemness of TNBC cells. It also improved the antitumor efficacy of paclitaxel in both TNBC cells and xenograft models. Mechanistically, honokiol may directly target the TAZ–TEAD complex, as indicated by molecular docking analysis. This interaction led to decreased YAP/TAZ protein levels and blockade of their nuclear accumulation, thereby suppressing the downstream transcriptional targets ANKRD1 and CYR61 in TNBC cells. **Conclusion:** Collectively, our findings suggest that honokiol is a promising therapeutic agent against TNBC, acting at least in part by suppressing the transcriptional activity of the YAP/TAZ–TEAD complex, thus attenuating cancer stemness and overcoming chemoresistance. These findings highlight honokiol’s translational potential.

## 1 Introduction

Triple-negative breast cancer (TNBC) remains a formidable clinical obstacle, largely driven by its aggressive phenotype and dismal prognosis [1]. The absence of hormone receptor expression and HER2 amplification precludes response to endocrine or HER2 targeted drugs, with chemotherapy serving as the standard systemic therapy [2,3,4]. Nevertheless, chemotherapeutic efficacy is frequently undermined by acquired resistance, which drives disease recurrence and worsens long-term survival [5,6]. Hence, there is an urgent need for novel agents that overcome resistance, thereby enhancing treatment efficacy and prolonging survival in these patients. YAP and TAZ serve as crucial downstream co-activators within the Hippo signaling axis. Accumulating evidence suggests that dysregulated YAP/TAZ signaling promotes tumor development, progression, invasion, and metastasis, while also driving resistance to chemotherapy, targeted therapy, and radiotherapy [7,8,9,10,11]. YAP/TAZ-driven therapy resistance is mediated by multiple key mechanisms, including enhanced cell survival, maintenance of cancer stem cells (CSCs), induction of epithelial–mesenchymal transition (EMT), upregulation of drug efflux pumps, and remodeling of the tumor microenvironment (TME) [12,13]. Recent research on TNBC provides evidence that targeting YAP/TAZ can overcome chemoresistance [14,15], positioning YAP/TAZ as a promising candidate for countering this resistance.

Studies have revealed that natural products can target YAP/TAZ, thereby reversing chemotherapy resistance in various tumors [16]. Honokiol, which is a phytochemical derived from the traditional Chinese herb *Magnolia officinalis*, potently suppresses tumor proliferation and neovascularization via multiple signaling mechanisms [17,18]. The objective of this study was to examine whether honokiol could inhibit CSC properties and improve paclitaxel sensitivity in TNBC, and to explore its mechanism of action. Our findings show that honokiol suppresses the CSC properties and enhances sensitivity to paclitaxel chemotherapy in TNBC. Mechanistically, honokiol reduces the protein levels of YAP/TAZ and their transcriptional partner TEAD, thereby downregulating the downstream oncogenic targets ANKRD1 and CYR61. Molecular docking suggests that honokiol may directly target the TAZ–TEAD complex. Hence, combining honokiol with paclitaxel represents a promising strategy, contributing to its further development as a novel therapy for TNBC.

## 2 Materials and Methods

### 2.1 Cell Lines and Reagents

The human-derived TNBC cell lines MDA-MB-231 (#SCSP-5043) and MDA-MB-468 (#SCSP-5053), the murine TNBC cell line 4T1 (#SCSP-5056), and the non-malignant breast epithelial cell line MCF10A (#SCSP-575) were obtained from Shanghai Cell Bank, Chinese Academy of Sciences (Shanghai, China). All cell lines were authenticated and confirmed to be mycoplasma-negative through routine testing. MCF10A cells were cultured as previously described [19]. MDA-MB-468 and MDA-MB-231 cells were maintained in high glucose Dulbecco’s Modified Eagle Medium (DMEM) (VivaCell, #C3113, Shanghai, China), whereas 4T1 cells were cultured in RPMI 1640 medium (VivaCell, #C3010, Shanghai, China). All media were supplemented with 1% antibiotic mixture (Gibco, #15140122, Waltham, MA, USA) and 10% fetal bovine serum (Gibco, #10270-106, Waltham, MA, USA). Cells were cultured under humidified conditions (37°C, 5% CO_2_). Honokiol (#S2310, purity > 99%) and paclitaxel (#T0968) were purchased from Selleckchem (Houston, TX, USA) and TargetMol (Wellesley Hills, MA, USA), respectively. Dimethyl sulfoxide (DMSO) (#196055) was obtained from MP Biomedicals (Santa Ana, CA, USA). In honokiol-treated cells, the DMSO concentration was maintained at 0.1% (v/v). Sulforhodamine B (SRB) (#230162) was supplied by Sigma (Burlington, MA, USA).

### 2.2 Dual-Luciferase Report Assay

MDA-MB-231 cells were co-transfected with two reporter plasmids using Lipofectamine 3000 (Thermo Fisher Scientific, #L3000 015, Waltham, MA, USA): 100 ng of the 8× GTIIC-luciferase construct (Addgene #34615, a gift from Dr. Stefano Piccolo), which contains a synthetic promoter responsive to YAP/TAZ, and 10 ng of the pRL-TK Renilla vector (Promega, #E2241, Madison, WI, USA). After a 24-h recovery period, the transfected cells were treated with honokiol (10, 20, and 40 μM) or DMSO vehicle for an additional 24 h. We then quantified dual luciferase (firefly and Renilla) activities using Promega’s kit (Promega, #E1910, Madison, WI, USA) according to the standard protocol, normalized firefly luminescence to Renilla luciferase activity, and converted the resulting ratios to fold changes relative to the DMSO control. Three independent biological replicates were performed, each with three technical replicates.

### 2.3 Cell Growth Analysis

For SRB cell growth assay, 6000 cells/well were seeded into 96-well dishes, followed by a 24-h adhesion period. We then treated the cells with honokiol (0, 5, 10, 20, 40, and 80 μM) for 24, 48, or 72 h. We fixed the incubated cells with 10% trichloroacetic acid (TCA) at 4°C for 1 h and then stained them with 0.4% (w/v) SRB solution. We solubilized the protein-bound dye in Tris base and recorded the optical density at 515 nm (reference: 690 nm) using an Enspire microplate reader (PerkinElmer, Inc., Waltham, MA, USA). After subtracting blank readings, we normalized the absorbance values against the untreated controls and presented them as percentage viability. We carried out this experiment in three biological replicates, each comprising five technical replicates. Using GraphPad Prism 8 (GraphPad Software, Inc., San Diego, CA, USA), we determined the 72-h IC50 values by nonlinear regression.

We assessed the pharmacological interaction between honokiol and paclitaxel using the Chou-Talalay method and computed the combination index (CI) with CompuSyn software (ComboSyn, Inc., Paramus, NJ, USA) [20]. Drug combinations were designed at fixed-ratio dilutions. For MDA-MB-231 cells, we combined Honokiol (HNK, 10 and 20 μM) with paclitaxel (PTX, 1.56, 3.125, 6.25, and 12.5 nM); for MDA-MB-468 cells, we combined HNK (15, 30 μM) with PTX (0.3125, 0.625, 1.25, 2.5 nM). We calculated the CI using averaged data from at least three independent experiments and considered a CI < 1 as synergistic.

For the colony formation assay, MDA-MB-231 and MDA-MB-468 cells were seeded into 6-well plates at 1000 cells per well and exposed to honokiol (20 μM and 30 μM, respectively), paclitaxel (6.25 nM and 2.5 nM, respectively), or their combination for 3 days. Colonies were then allowed to proliferate in drug-free medium for an additional 7–10 days. After incubation, the colonies were fixed and stained with 4% paraformaldehyde (Biosharp, BL539A, Beijing, China) and 0.1% crystal violet, respectively (Amresco, #0528, Solon, OH, USA). Colonies containing ≥50 cells were scored manually under a microscope. The survival fraction was calculated relative to the untreated control and expressed as a percentage.

### 2.4 Tumorsphere Formation Assay

To determine the activity of honokiol against CSC-enriched populations, MDA-MB-231 cells were subjected to 10 or 20 μM honokiol, and MDA-MB-468 and 4T1 cells to 15 or 30 μM honokiol, all for 72 h. The treated cells were then resuspended in serum-free tumorsphere medium consisting of DMEM/F12 (Gibco, 11320033, Waltham, MA, USA) supplemented with 1× B27 (Gibco, 17504-044, Waltham, MA, USA), 20 ng/mL EGF (PeproTech, AF-100-15, Rocky Hill, NJ, USA), and 20 ng/mL bFGF (PeproTech, 100-18B, Rocky Hill, NJ, USA), and were plated into ultra-low-attachment 6-well plates (Corning, 3471, NY, USA) at 10,000 cells per well, with DMSO-only controls always included. This suspension culture enabled spheroid formation and preserved stemness, a well-recognized method in cancer stem cell research. On day 10, the cultures were visualized under a Zeiss inverted microscope (Axio Vert.A1, Carl Zeiss AG, Oberkochen, Germany) and all spheres larger than 50 μm were counted.

### 2.5 RNA Isolation and Quantitative RT-PCR

We isolated RNA from cultured cells with Trizol reagent (Invitrogen, #15596026, Carlsbad, CA, USA), and then evaluated its purity and concentration by measuring the A260/A280 ratio (≥1.8) on a NanoDrop™ spectrophotometer (Thermo Fisher Scientific, Waltham, MA, USA). Using oligo(dT) and M-MuLV reverse transcriptase (Thermo Fisher, #28025013, Waltham, MA, USA), we generated cDNA from 1 μg of total RNA per sample in 20-μL reactions. qPCR was then conducted on a Bio-Rad CFX96 instrument with Power SYBR Green Master Mix (Roche, #04887352001, Mannheim, Germany). Amplification was carried out using a 10-min denaturation at 95°C, then 40 two-step cycles of 95°C for 15 s and 60°C for 60 s. Following amplification, a melting curve analysis was carried out to confirm the specificity of the intended PCR amplicons. We performed three independent biological replicates per condition, with each sample subjected to technical triplicate analysis. β-actin was used as the normalization reference, and relative mRNA levels were then determined by the 2^−ΔΔCt^ method.

Table 1 lists the primer sequences in the 5′-to-3′ orientation.

**Table 1:** The primers used in RT-qPCR.

Genes	Forward Primer (5′–3′)	Reverse Primer (5′–3′)
h YAP	TCCTGATGGATGGGAACAAG	ATGGCAAAACGAGGGTCA
h TAZ	CAGCAATGTGGATGAGATGG	TCAAGGAAATCAGGGAAACG
h ANKRD1	AGTAGAGGAACTGGTCACTGG	TGTTTCTCGCTTTTCCACTGTT
h CTGF	GCAGAGCCGCCTGTGCATGG	GGTATGTCTTCATGCTGG
h CYR61	CACACCAAGGGGCTGGAATG	CCCGTTTTGGTAGATTCTGG
h β-Actin	CCAACCGCGAGAAGATGA	CCAGAGGCGTACAGGGATAG
m YAP	TGAGATCCCTGATGATGTACCAC	TGTTGTTGTCTGATCGTTGTGAT
m TAZ	GAAGGTGATGAATCAGCCTCTG	GTTCTGAGTCGGGTGGTTCTG
m ANKRD1	GGAACAACGGAAAAGCGAGAA	GAAACCTCGGCACATCCACA
m CTGF	GACCCAACTATGATGCGAGCC	CCCATCCCACAGGTCTTAGAAC
m CYR61	TAAGGTCTGCGCTAAACAACTC	CAGATCCCTTTCAGAGCGGT
m β-Actin	GGCTGTATTCCCCTCCATCG	CCAGTTGGTAACAATGCCATGT

### 2.6 Western Blot Analysis

We lysed cells in RIPA buffer supplemented with protease inhibitors (Thermo Fisher Scientific, 1862209, Waltham, MA, USA) and measured protein concentrations using a BCA assay kit (Thermo Fisher Scientific, Waltham, MA, USA). Equal amounts of protein (20 μg/lane) were then resolved on 10% SDS-PAGE and analyzed by immunoblotting. Primary antibodies against YAP (#4912), TAZ (#70148), TEADs (#13295), GAPDH (#2118), and CYR61 (#14479) (Cell Signaling Technology, Danvers, MA, USA), as well as ANKRD1 (#A6192), CD133 (#A0219), Nestin (#A11861), and OCT4 (#A7920) (Abclonal, Woburn, MA, USA) were diluted at 1:1000 in TBST containing 5% BSA and incubated overnight at 4°C. Secondary antibodies consisted of HRP-conjugated anti-mouse (#1706516) and HRP-conjugated anti-rabbit (#1706515), both from Bio-Rad (Hercules, CA, USA). We detected protein bands using ECL detection reagents (Thermo Fisher Scientific, #34580, Waltham, MA, USA) and visualized them with a chemiluminescence detection system (Tanon-660, Shanghai Tanon Technology Co., Ltd., Shanghai, China). GAPDH was used as the loading control.

### 2.7 Immunofluorescence Staining

We plated TNBC cells in 35-mm dishes with glass bottoms (NEST, Wuxi, China). After adhesion, cells were exposed to honokiol at the specified concentrations for 72 h. Immunofluorescence staining was then carried out according to a standard protocol. Briefly, cells were fixed in 4% paraformaldehyde (Biosharp, #BL539A, Beijing, China) for 15 min, permeabilized with 0.1% Triton X-100 (Solarbio, #T8200, Beijing, China) for 10 min, and blocked with 5% BSA (Solarbio, #A8010, Beijing, China) for 1 h at RT. We then incubated the samples overnight at 4°C with primary antibodies against YAP (#A1002SP) and TAZ (#A8202). After three PBS washes, the cells were incubated with Alexa Fluor™ 488-conjugated goat anti-rabbit secondary antibody (1:200, Thermo Fisher Scientific, #A27034) for 1 h at RT in the dark. Nuclei were counterstained with DAPI (Solarbio, #C0060, Beijing, China), and coverslips were mounted with Prolong™ Gold Antifade Mountant (Invitrogen, #P10144, Carlsbad, CA, USA). We then captured the fluorescent images with a fluorescence microscope (Carl Zeiss, LSM800, Jena, Germany).

### 2.8 Tumor Xenograft Experiment

All animal experiments were approved by the Animal Ethics Committee of Guangzhou University of Traditional Chinese Medicine. (Approval No.: 20220310009). We purchased 16 female BALB/c nude mice (6 weeks old, 18–20 g) from Charles River (Beijing, China). Mice were housed in a specific pathogen-free (SPF) setting, provided with food and water without restriction, and underwent a 7-day acclimation period prior to experiment. Mice were inoculated subcutaneously in the right anterior axilla with 1 × 10^6^ 4T1 cells resuspended in 100 μL of PBS. After xenograft tumors reached approximately 50 mm^3^, mice were randomly assigned to four groups (n = 4/group) by a computer-generated random number sequence. The groups received intraperitoneal injections for 21 days as follows: saline (vehicle control), paclitaxel alone (5 mg/kg on alternate days), honokiol alone (20 mg/kg daily), or the combination regimen consisting of honokiol (20 mg/kg daily) plus paclitaxel (5 mg/kg on alternate days). All drugs were freshly prepared and administered between 09:00 and 11:00 daily. Tumor volume was measured every 3 days by an investigator blinded to group allocation using a caliper, and calculated as volume (mm^3^) = length × width^2^/2. Predefined humane endpoints included tumor volume >2000 mm^3^, tumor ulceration/necrosis, >20% decrease from baseline body weight, and loss of access to food or water. No animals were excluded or died during the experiment; all 16 mice were included in the final analysis (n = 4 per group). After treatment, tumors were collected, weighed, photographed, and subsequently embedded in paraffin for immunohistochemical analysis. The investigator remained blinded to group allocation until statistical analysis was completed.

### 2.9 Immunohistochemistry

For immunohistochemical staining, formalin-fixed, paraffin-embedded tumor sections were deparaffinized in xylene and rehydrated through a graded ethanol series. Antigen retrieval was performed by heating sections in 10 mM sodium citrate buffer (pH 6.0) (Servicebio, #G1202, Wuhan, China) in a pressure cooker for 15 min, followed by cooling to RT. Endogenous peroxidase activity was blocked with 3% H_2_O_2_ in methanol for 10 min. After washing with PBS, the sections were blocked with 5% BSA and incubated overnight at 4°C with primary antibodies against YAP (1:200, Abclonal, #A1002SP, Woburn, MA, USA), TAZ, (1:50, Abclonal, #A8202, Woburn, MA, USA), ANKRD1 (1:200, Proteintech, #11427-1-AP, Wuhan, China), and Ki67 (1:200; Invitrogen, #PA5-19462, Carlsbad, CA, USA) overnight at 4°C. Following thorough washing, the sections were incubated with HRP-conjugated secondary antibody (Servicebio, #GB23303, Wuhan, China) for 1 h at RT. Immunoreactivity was visualized with DAB (Servicebio, #G1212, Wuhan, China), and sections were counterstained with hematoxylin (Servicebio, #G1004, Wuhan, China). We then dehydrated, cleared, mounted the slides with neutral resin (Servicebio, #G8590, Wuhan, China), and captured images using an OLYMPUS VS200 system (Tokyo, Japan). Relative optical density (ROD) of IHC signals was quantified using ImageJ from at least four random fields per section, normalized to the control (set as 1.0), and presented as fold changes.

### 2.10 Molecular Docking Analysis

The three dimensional (3D) structure of honokiol was retrieved from PubChem (CID: 72303) and converted to a 3D model using OpenBabel (v2.4.1). Geometry optimization was performed via 500 steps of steepest-descent minimization with the MMFF94 force field. The optimized ligand was imported into AutoDockTools 1.5.6, where non polar hydrogens were merged, Gasteiger partial charges were assigned, rotatable bonds were automatically detected, and default torsion parameters were retained. The prepared ligand was exported in PDBQT format.

The TAZ–TEAD complex structure (PDB: 5GN0) was downloaded from the Protein Data Bank (https://www.rcsb.org/). Receptor preparation involved removing all crystallographic water molecules and heteroatoms, adding polar hydrogens, and assigning Kollman united-atom charges using AutoDockTools 1.5.6; all side chains were kept rigid throughout the docking procedure.

A 60 × 60 × 60 grid box (0.375 Å spacing) was centered on the co-crystallized ligand’s center, ensuring the box fully encompassed the entire binding cleft with a 2.90 Å margin around key active-site residues. Docking was performed using the Lamarckian Genetic Algorithm (LGA) with the following parameters: 100 independent docking runs, an initial population of 150, a maximum of 2,500,000 energy evaluations, 27,000 generations, a mutation rate of 0.02, and a crossover rate of 0.80.

Docking poses were ranked by the lowest estimated free energy of binding (ΔG), and clustering was performed with a root mean square deviation (RMSD) threshold of 2.0 Å. ΔG values (kcal/mol) were computed using AutoDock’s semi empirical scoring function, which integrates van der Waals, electrostatic, hydrogen bond, desolvation, and entropy components. Within this framework, a lower ΔG corresponds to higher predicted binding affinity. Binding interactions were visually analyzed and detailed using PyMOL 2.5.3 (Schrödinger) and LigPlus 2.2 (EMBL EBI).

### 2.11 Statistical Analysis

Data are presented as mean ± SD from at least three independent experiments. Data normality distribution was verified by the Shapiro–Wilk test, and variance homogeneity was assessed prior to statistical analysis. Two-group comparisons were conducted via Student’s two-tailed *t*-test, while multiple-group differences were assessed using one-way ANOVA followed by Tukey’s post hoc test. Statistical significance was set at *p* < 0.05, with n representing the number of biological replicates or animals per group.

## 3 Results

### 3.1 Honokiol Inhibited Cell Proliferation in Triple-Negative Breast Cancer

**Figure 1:**
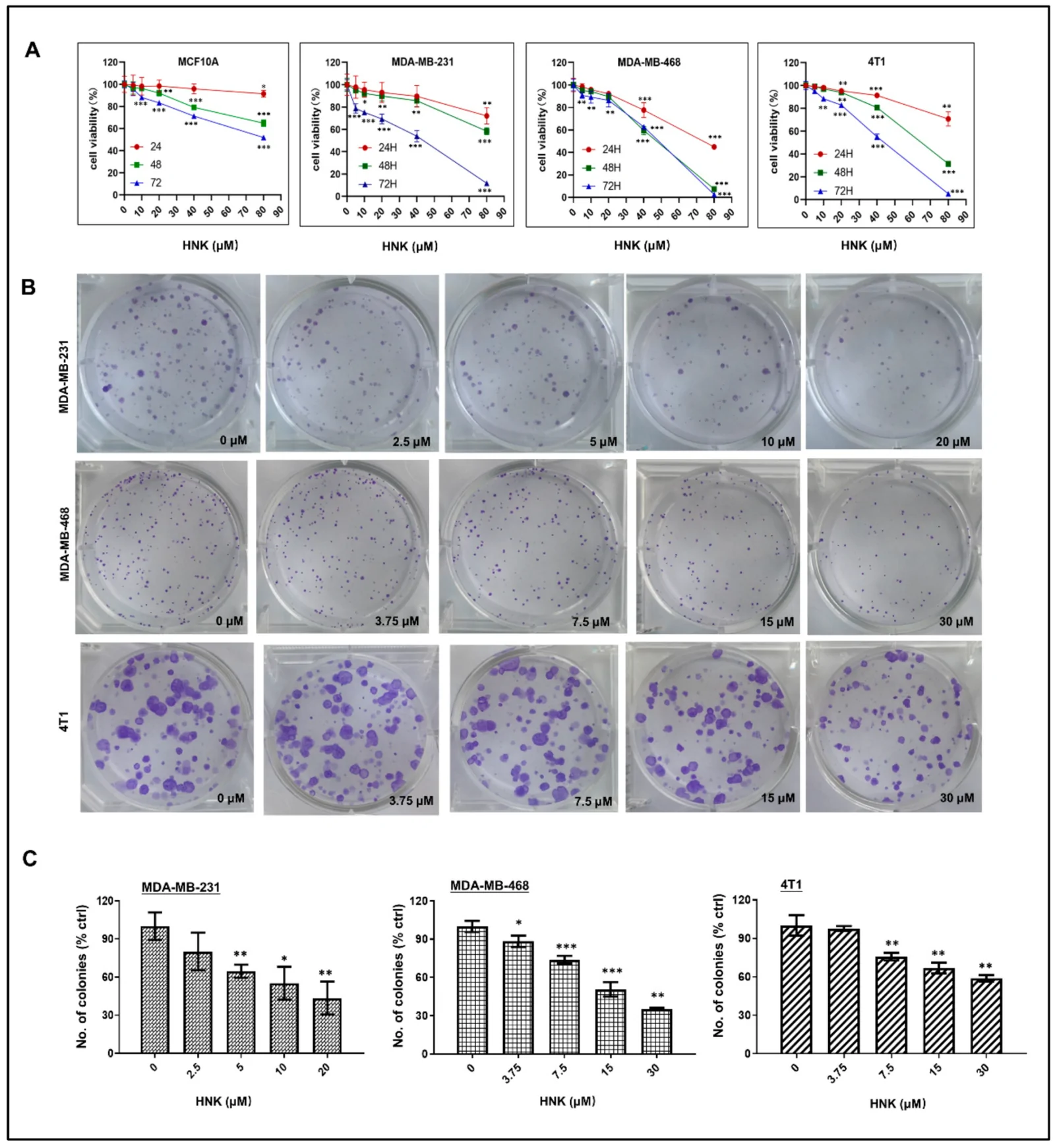
Honokiol inhibited cell proliferation in triple-negative breast cancer. (**A**) MCF10A and three TNBC cell lines (MDA-MB-468, MDA-MB-231, and 4T1) were treated with 0–80 μM honokiol (HNK) for 24–72 h, after which cell survival was assessed. The 72-h IC50 values were calculated using GraphPad Prism. (**B**) Representative images of colony formation are shown. These TNBC cell lines were seeded in six-well plates, incubated with the given doses of honokiol for 3 days, and then cultured in honokiol-free medium. Ten days later, colonies were visualized with 0.1% crystal violet and quantified. (**C**) Colonies were scored in at least three independent experiments per cell line. All values are reported as the mean ± SD. Statistical significance was defined as *p < 0.05, **p < 0.01, and ***p < 0.001.

To assess the cytotoxicity of honokiol (HNK), we performed SRB assays on MCF10A (non-tumorigenic mammary epithelial cell line) and three TNBC cell lines (MDA-MB-468, MDA-MB-231, and 4T1). The data showed that an IC50 of 89.39 μM at 72 h was obtained for MCF10A cells (Fig. 1A), indicating that non-cancerous cells showed lower susceptibility to honokiol than the TNBC lines. Honokiol potently suppressed the proliferation of all three TNBC cell lines depending on both concentration and exposure time, yielding 72-h IC50 values of 31.39 μM (MDA-MB-231), 43.89 μM (MDA-MB-468), and 41.65 μM (4T1). In addition, colony formation assays showed that honokiol effectively suppressed colony formation in all three TNBC cell lines, indicating its long-term inhibitory effects (Fig. 1B,C).

### 3.2 Honokiol Inhibited YAP/TAZ–TEAD Complex Activity in Triple-Negative Breast Cancer

**Figure 2:**
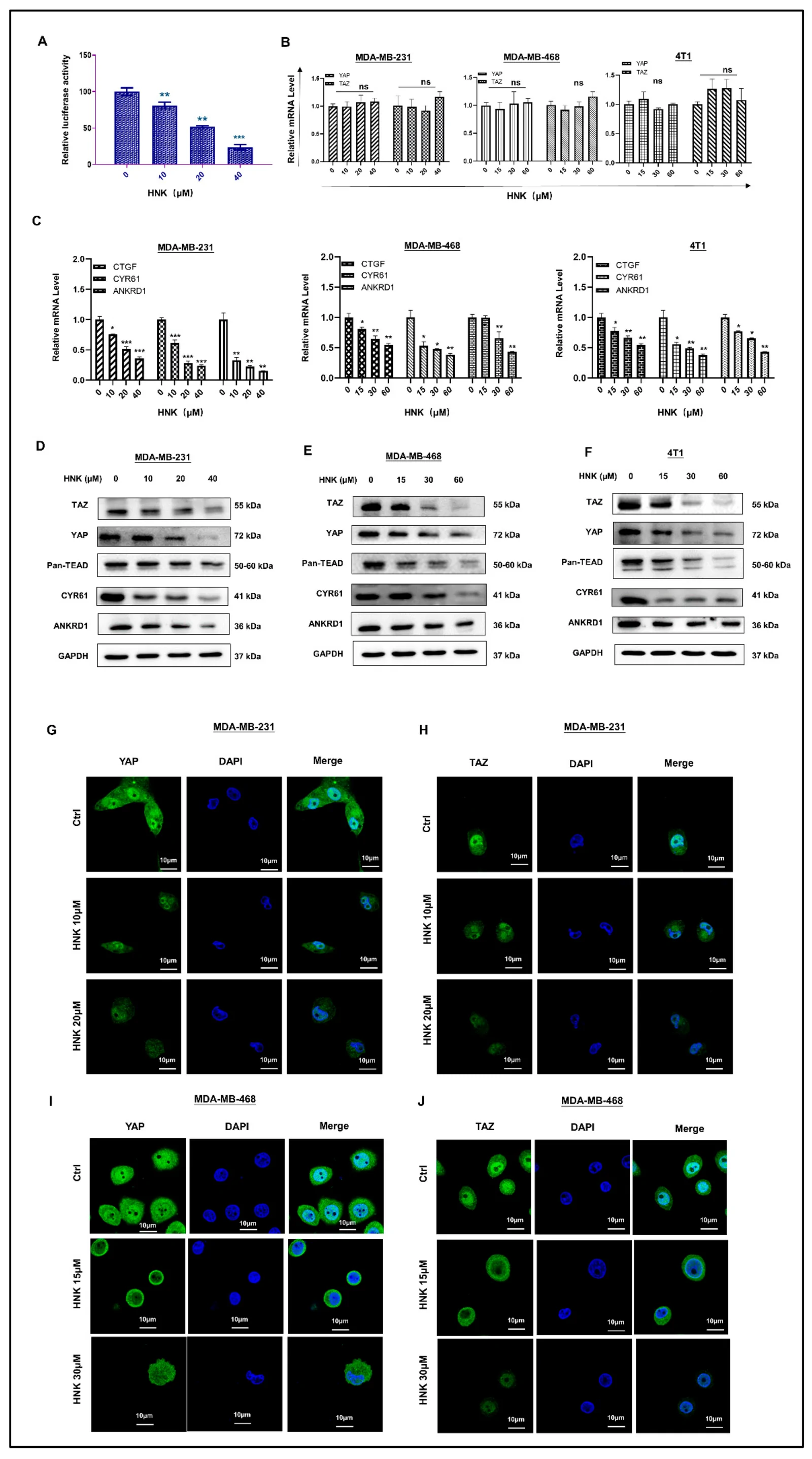
Honokiol inhibited YAP/TAZ–TEAD complex activity in triple-negative breast cancer. (**A**) The 8xGTIIC-luciferase reporter and pRL-TK were co-transfected into MDA-MB-231 cells, followed by honokiol (HNK) treatment for 24 h. Firefly luciferase signals were adjusted by Renilla luciferase activity, and the results were expressed as a percentage compared with the control values. (**B**) The transcript levels of YAP and TAZ were measured using RT-qPCR. The mean ± SD from three replicate experiments is reported for all data. ns, no significance. (**C**) CTGF, CYR61, and ANKRD1 mRNA levels were quantified by RT-qPCR. (**D**–**F**) Protein expression levels of YAP, TAZ, TEAD, CYR61, and ANKRD1 were determined by Western blotting in MDA-MB-231 (**D**), MDA-MB-468 (**E**), and 4T1 (**F**) cells following 72-h exposure to honokiol. GAPDH served as the internal control. (**G**–**J**) Immunofluorescence revealed YAP/TAZ (green) cellular localization in MDA-MB-231 (**G**,**H**) and MDA-MB-468 (**I**,**J**) cells. DAPI (blue) was used for nuclear counterstaining. Representative images are presented from at least three independent experiments. Scale bar: 10 μm. Data are shown as mean ± SD (n = 3). *p < 0.05, **p < 0.01, ***p < 0.001, ns no significance.

We first observed that honokiol dose-dependently inhibited YAP/TAZ reporter activity in MDA-MB-231 cells, indicating YAP/TAZ-mediated transcription suppression (Fig. 2A). Next, we assessed its effects on endogenous YAP/TAZ–TEAD transcriptional targets. Our results demonstrated that honokiol suppressed the mRNA levels of ANKRD1, CTGF, and CYR61, without altering those of TAZ or YAP in three TNBC cell lines (Fig. 2B,C). Immunoblotting showed that honokiol downregulated the protein abundance of YAP, TAZ, TEADs, CYR61, and ANKRD1 in TNBC cells (Fig. 2D–F). As nuclear localization is a prerequisite for YAP/TAZ function, we next examined their subcellular distribution. Immunofluorescence staining showed that honokiol reduced nuclear YAP/TAZ accumulation (Fig. 2G–J).

### 3.3 Honokiol Inhibited the Stemness of Triple-Negative Breast Cancer Cells

**Figure 3:**
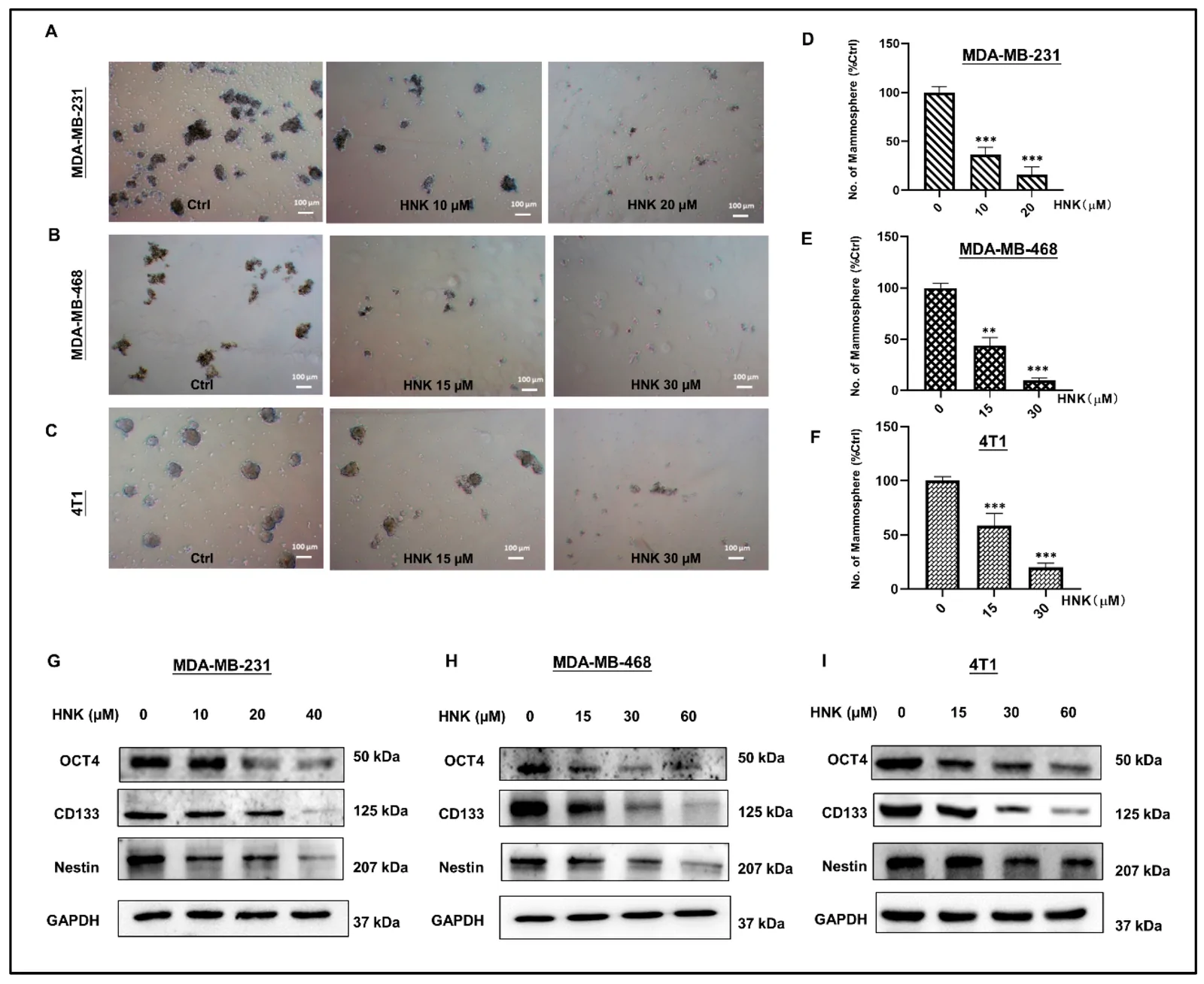
Honokiol inhibited the stemness of triple-negative breast cancer cells. (**A**–**C**) A decrease in both the number and size of primary tumorspheres was observed in MDA-MB-231 (**A**), MDA-MB-468 (**B**), and 4T1 (**C**) cells following honokiol (HNK) treatment. (scale bar = 100 μm). The assay was independently repeated three times for each TNBC cell line. Four random images per well were also acquired from arbitrarily selected regions. (**D**–**F**) Tumorsphere counts were measured and presented as percentages normalized to the untreated control in MDA-MB-231 (**D**), MDA-MB-468 (**E**), and 4T1 (**F**) cells. Statistical significance is indicated as **p < 0.01 and ***p < 0.001. (**G**–**I**) The protein abundance of OCT4, CD133, and Nestin in MDA-MB-231 (**G**), MDA-MB-468 (**H**), and 4T1 (**I**) cells were evaluated via Western blot analysis. At least three independent replicates were performed for each experiment, with GAPDH as the loading control.

As YAP/TAZ is essential for maintaining stem like properties and tumor initiating potential in TNBC [21], we evaluated the anti CSC effects of honokiol at sub cytotoxic concentrations. Honokiol significantly impaired the sphere-forming capacity of TNBC cells, as evidenced by a decrease in sphere number and size (Fig. 3A–F). In addition, honokiol downregulated the expression of CSC-associated marker expression (Fig. 3G–I). Collectively, these findings indicate that honokiol reduces the CSC fraction within TNBC cells *in vitro*.

### 3.4 Honokiol Sensitized Triple-Negative Breast Cancer Cells to Paclitaxel Treatment

**Figure 4:**
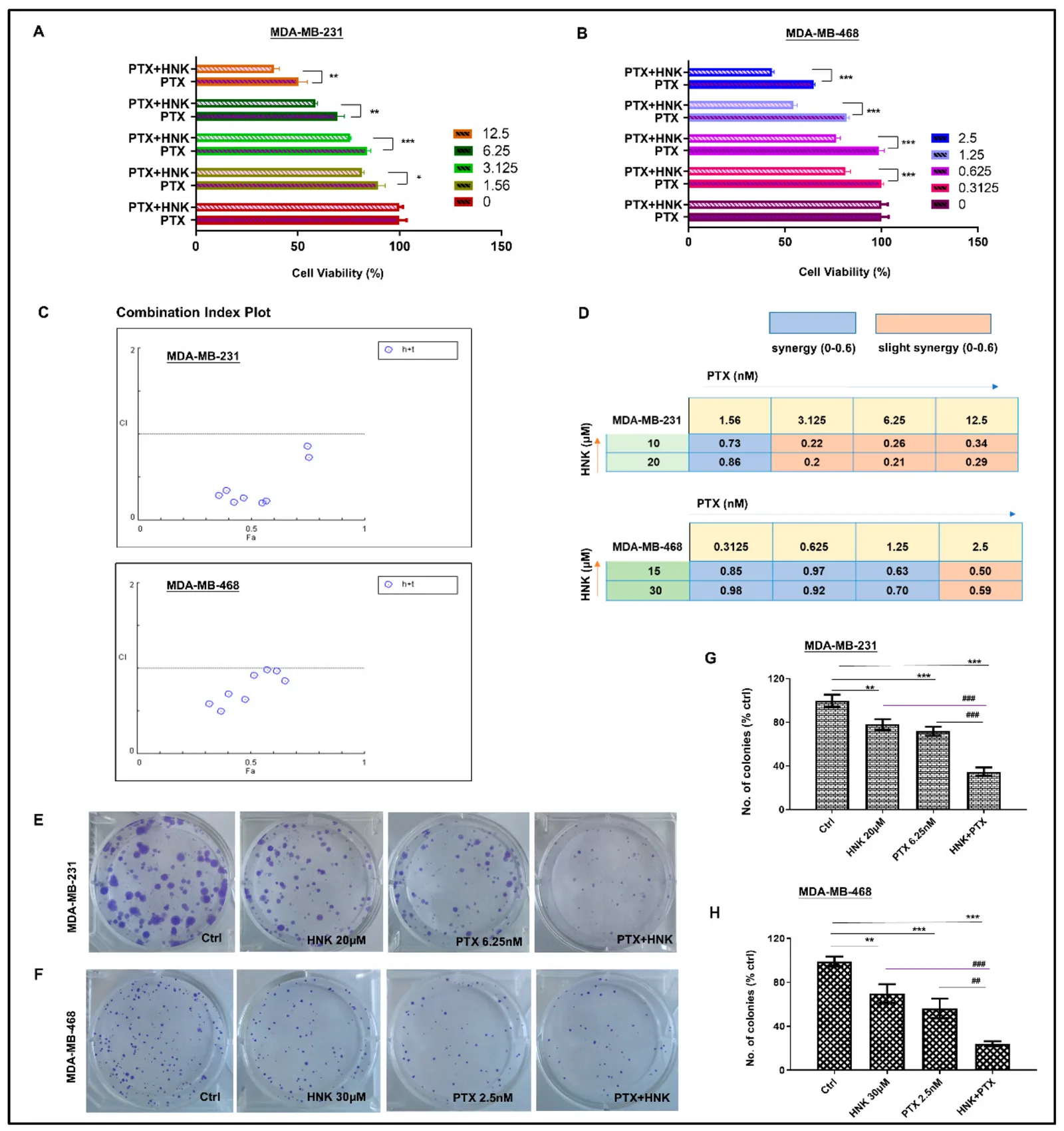
Honokiol sensitized triple-negative breast cancer cells to paclitaxel treatment. (**A**,**B**) MDA-MB-231 (**A**) and MDA-MB-468 (**B**) cells were cultured with elevated doses of paclitaxel (PTX), alone or combined with indicated honokiol (HNK) concentrations, for 72 h. Cell viability from SRB assay was presented as a percentage of the DMSO control. (**C**) The combination index (CI) plot in MDA-MB-231 and MDA-MB-468 cells are shown. (**D**) CI was calculated according to the Chou–Talalay method. (**E**,**F**) Representative images of colony formation in MDA-MB-231 (**E**) and MDA-MB-468 (**F**) cells are presented. (**G**,**H**) Colony quantification in MDA-MB-231 (**G**) and MDA-MB-468 (**H**) cells is expressed as mean ± SD (n = 3). *p < 0.05, **p < 0.01 and ***p < 0.001; ^##^*p* < 0.01 and ^###^*p* < 0.001.

**Figure A1:**
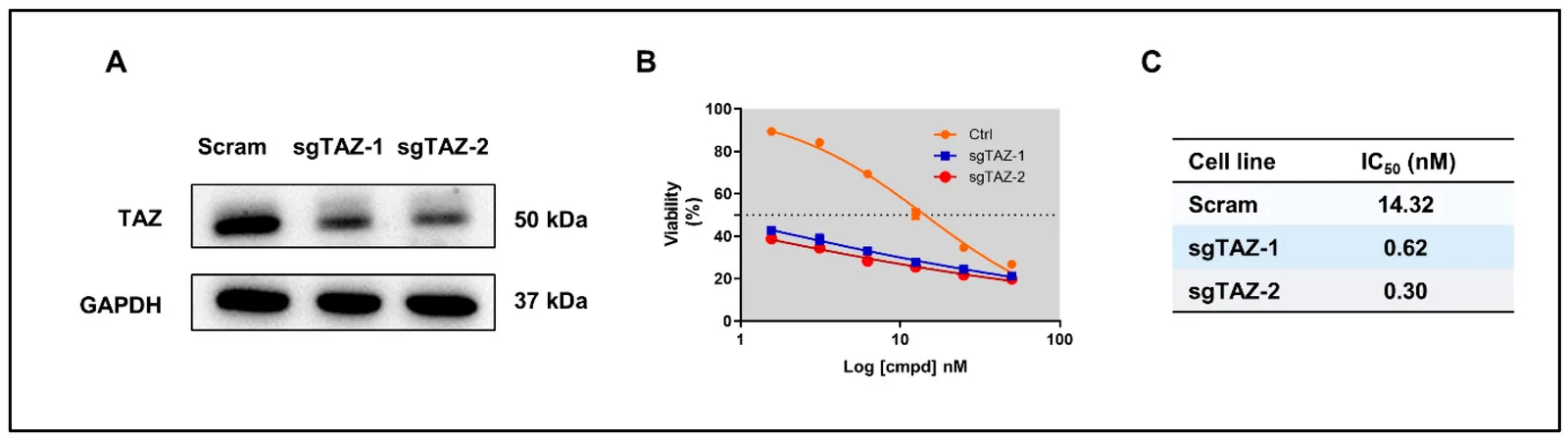
TAZ knockdown enhances the sensitivity of MDA-MB-231 cells to paclitaxel. (**A**) Validation of TAZ knockdown efficiency by Western blotting. (**B**) MDA-MB-231 cells were treated with escalating doses of paclitaxel for 72 h, followed by an SRB assay to determine cell viability. (**C**) The IC50 at 72 h was calculated using GraphPad Prism.

To further confirm that abnormal YAP/TAZ activation attenuates paclitaxel sensitivity in TNBC, we demonstrated that TAZ knockdown enhances paclitaxel-mediated anticancer activity in TNBC cells (Fig. A1). Subsequently, we evaluated honokiol’s potential to sensitize TNBC cells to paclitaxel using SRB and colony formation assays. Compared with paclitaxel monotherapy, the honokiol–paclitaxel combination significantly reduced cell viability in both TNBC cell lines tested (Fig. 4A,B). The combination exhibited synergistic effects, as evidenced by CI values less than 1 (Fig. 4C,D). Consistent with these findings, the honokiol–paclitaxel combination at sub-IC50 concentrations synergistically inhibited colony formation in both TNBC cell lines tested (Fig. 4E–H), demonstrating that honokiol potentiates paclitaxel sensitivity in TNBC cells.

### 3.5 The Honokiol–Paclitaxel Combination Exhibited Enhanced Inhibitory Effects against Triple-Negative Breast Cancer In Vivo

**Figure 5:**
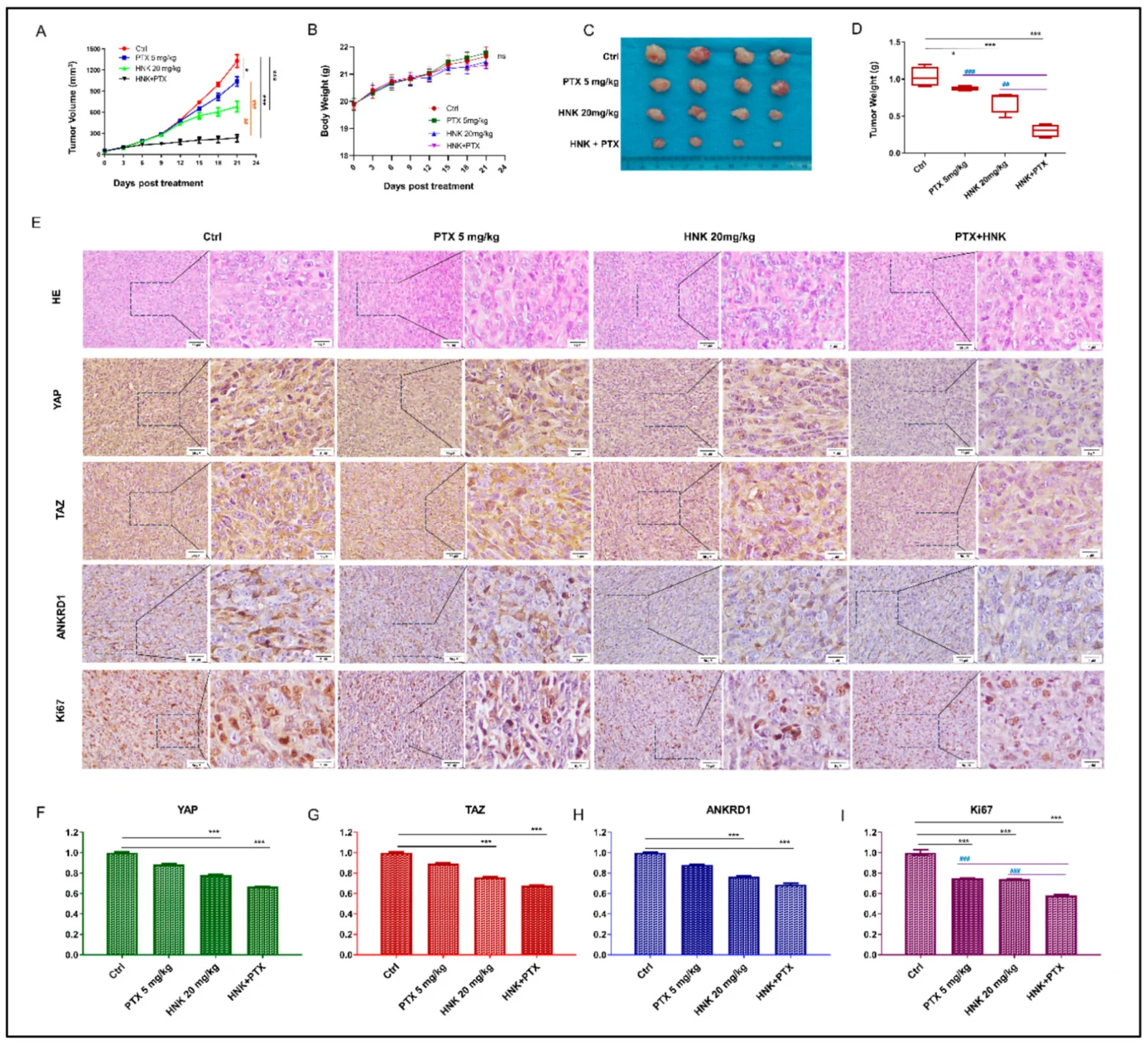
The honokiol–paclitaxel combination exhibited enhanced inhibitory effects against triple-negative breast cancer *in vivo*. BALB/c nude mice bearing 4T1 xenografts were treated intraperitoneally (i.p.) with vehicle, paclitaxel (PTX, 5 mg/kg every other day), honokiol (HNK, 20 mg/kg daily), or PTX + HNK in combination. (**A**) Tumor volumes were measured with calipers and calculated as (L × W^2^)/2. (**B**) Body weights were measured on the same schedule using a digital scale. Data are expressed as mean ± SD. (**C**) Tumor specimens were photographed against a metric ruler using a digital camera for scale indication. (**D**) Tumor weights were measured immediately after excision using a calibrated analytical balance. Data are shown as mean ± SD (n = 4 tumors/group, corresponding to 4 mice/group). (**E**) Immunohistochemical staining of YAP, TAZ, ANKRD1, and Ki67 in the designated TNBC xenograft tissues. Positive immunoreactivity was visualized as brown (DAB), while nuclei were counterstained blue with hematoxylin. Scale bars: 50 μm (left panels) and 5 μm (right panels). (**F**–**I**) Semiquantitative analysis of YAP (**F**), TAZ (**G**), ANKRD1 (**H**), and Ki67 (**I**) immunostaining in tumor tissue. The relative optical density (ROD) was quantified via ImageJ and expressed as fold change versus controls (normalized to 1.0). Values are expressed as mean ± SD (n = 4/group). *p < 0.05, ***p < 0.001; ^##^*p* < 0.01, ^###^*p* < 0.001; ns no significance.

To further explore honokiol’s *in vivo* effect on paclitaxel chemosensitivity in TNBC, we established nude mice bearing 4T1 xenografts. Co-administration of honokiol and paclitaxel yielded a significantly greater reduction in tumor volume than either honokiol or paclitaxel monotherapy (Fig. 5A). Moreover, all treatments were well tolerated, as evidenced by the absence of significant differences in mouse body weight (Fig. 5B). Consistently, the honokiol–paclitaxel combination significantly reduced the final tumor mass of the xenografts compared with the honokiol and the paclitaxel monotherapy groups (Fig. 5C,D). Using Ki67 IHC staining, we further assessed tumor proliferation and observed reduced activity in the co-treatment group (Fig. 5E,I). The antitumor effects were associated with the downregulation of YAP, TAZ, and ANKRD1 in xenograft tissues following honokiol treatment (Fig. 5E–H). These results confirm that honokiol enhances the efficacy of paclitaxel *in vivo*, supporting its potential in combination therapy for TNBC.

### 3.6 Honokiol Interacted with the TAZ–TEAD Complex

**Figure 6:**
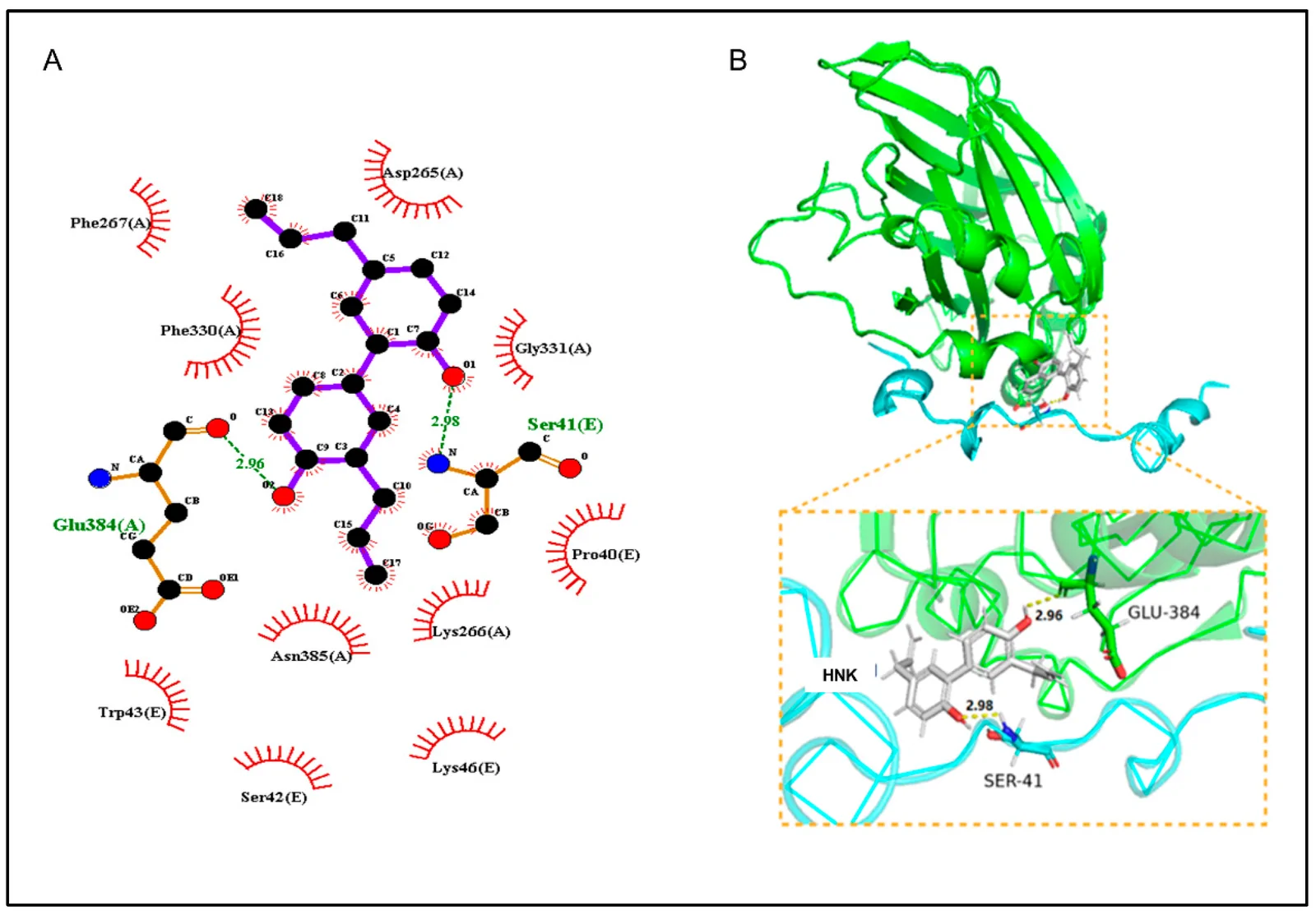
Honokiol interacted with the TAZ–TEAD complex. (**A**) Honokiol binds to the SER-41 and GLU-384 in the 2D model. (**B**) Honokiol (Grey) binds to the SER-41 (Blue) and GLU-384 (Green) with the H-bonds colored yellow in the 3D structure.

Honokiol was predicted by molecular docking to bind to the TAZ-TEAD complex, exhibiting a favorable energy score of −5.08 kcal/mol. Structural analysis showed that honokiol primarily interacts with TAZ, forming hydrogen bonds with Glu384 and Ser41 and engaging in hydrophobic interactions within a pocket composed of residues Phe267, Asp265, Phe330, Gly331, Pro40, Lys266, Asn385, Trp43, Ser42, and Lys46. These interactions stabilize binding, allowing honokiol’s hydrophobic moiety to be tightly embedded within the complex’s cavity (Fig. 6A,B).

## 4 Discussion

Aberrant activation of YAP/TAZ is closely correlated with TNBC aggressiveness, making YAP/TAZ a promising therapeutic target [22,23]. This study presents evidence that honokiol treatment leads to a concentration-dependent decrease in YAP and TAZ protein abundance in TNBC. YAP/TAZ oncogenic functions rely mainly on nuclear localization and complexation with TEAD transcription factors, which in turn activate downstream target genes such as ANKRD1 and CYR61, thereby driving core cancer hallmarks [24,25,26,27]. The study further confirmed that honokiol treatment markedly reduces the abundance of TEAD and its well-established downstream mediators, CYR61 and ANKRD1. Coordinated downregulation of the coactivators (YAP/TAZ) and their DNA-binding partner (TEAD) suggests that honokiol acts as a multifaceted inhibitor of this signaling pathway.

The persistence of CSCs and the development of chemoresistance are two key hallmarks of TNBC progression. CSCs are the main drivers of TNBC initiation, progression, recurrence, and chemoresistance [28,29]. The YAP/TAZ–TEAD signaling pathway is a key regulator of CSC maintenance [30,31,32]. Paclitaxel is a standard chemotherapeutic agent for TNBC; nevertheless, its clinical effectiveness is often limited by the emergence of resistance [33]. Recent findings suggest that activation of the YAP/TAZ–TEAD signaling pathway drives paclitaxel resistance in TNBC [34,35]. This study shows that honokiol simultaneously targets the two hallmarks of TNBC progression through disrupting the YAP/TAZ–TEAD axis. Honokiol suppresses the sphere-forming ability of TNBC cells while downregulating CSC markers. Furthermore, honokiol significantly enhanced the cytotoxic effects of paclitaxel against TNBC cell lines, as evidenced by reduced cell viability. Moreover, *in vivo* xenograft models revealed that the honokiol–paclitaxel combination led to a greater reduction in tumor volume and in Ki67 proliferation marker expression than monotherapy with either agent. Honokiol, which has a well-established safety profile in preclinical models, has been recently under investigation in early-phase clinical trials for other cancers [36]. These findings carry significant clinical implications and suggest that honokiol may be combined with paclitaxel to enhance treatment outcomes for patients with TNBC, particularly those with paclitaxel-resistant tumors.

Given that honokiol decreased YAP/TAZ protein expression but did not affect their transcript levels, these results suggest a post-transcriptional regulatory mechanism. Molecular docking analysis further indicated honokiol’s potential interaction with the TAZ–TEAD complex. Such an interaction may confer specificity benefits, especially because TAZ generally shows higher expression than YAP in TNBC and is associated with significantly worse clinical outcomes [37].

This study is subject to several limitations. First, evidence that honokiol directly targets the TAZ–TEAD complex is currently preliminary, as molecular docking analysis did not show strong binding affinity, and no biochemical assays (e.g., co-IP or NanoBiT) have confirmed direct disruption of the interaction. Second, the absence of a YAP/TAZ rescue assay precludes the establishment of a definitive causal link between YAP/TAZ downregulation and the observed anticancer effects. Third, although honokiol reduces YAP/TAZ protein levels without affecting their mRNA expression, the exact post-transcriptional mechanism underlying this discrepancy remains unclear. Thus, while honokiol shows promise against TNBC, its precise mechanism warrants further validation. Nevertheless, the functional data support honokiol’s potential as a therapeutic agent against TNBC.

Collectively, our findings demonstrate that honokiol not only suppresses CSC function but also enhances the cytotoxicity of paclitaxel in both TNBC cell lines and xenograft models. The underlying mechanism may be related to honokiol’s ability to reduce YAP/TAZ–TEAD complex activity, thereby overcoming drug resistance. Therefore, honokiol–paclitaxel combination therapy may be a promising clinical strategy for TNBC treatment.

## Data Availability

The raw data supporting the findings of this study are available from the corresponding author upon reasonable request.

## Abbreviations

TNBC	triple-negative breast cancer
CSCs	cancer stem cells
EMT	epithelial-mesenchymal transition
TME	tumor microenvironment
FBS	fetal bovine serum
SRB	sulforhodamine B
TCA	trichloroacetic acid
IC50	half-maximal inhibitory concentration
DMSO	dimethyl sulfoxide
PVDF	polyvinylidene difluoride
CI	combination index
SD	standard deviation
ULAPs	ultra-low-attachment plates
EGF	epidermal growth factor
bFGF	basic fibroblast growth factor
SPF	specific pathogen-free
ROD	relative optical density
